# On standardized relative survival

**DOI:** 10.1111/biom.12578

**Published:** 2016-08-23

**Authors:** Peter Sasieni, Adam R. Brentnall

**Affiliations:** ^1^ Centre for Cancer Prevention, Wolfson Institute of Preventive Medicine, Queen Mary University of London, Charterhouse Square London, EC1M 6BQ, U.K.

**Keywords:** Cancer, Ederer‐II, Epidemiology, Excess Hazards, Heterogeneity, Net Survival, Relative Survival, Robust Estimation, Standardization, Weighted Excess Hazards, Weighted Net Survival, Weighted Relative Survival

## Abstract

Cancer survival comparisons between cohorts are often assessed by estimates of relative or net survival. These measure the difference in mortality between those diagnosed with the disease and the general population. For such comparisons methods are needed to standardize cohort structure (including age at diagnosis) and all‐cause mortality rates in the general population. Standardized non‐parametric relative survival measures are evaluated by determining how well they (i) ensure the correct rank ordering, (ii) allow for differences in covariate distributions, and (iii) possess robustness and maximal estimation precision. Two relative survival families that subsume the Ederer‐I, Ederer‐II, and Pohar‐Perme statistics are assessed. The aforementioned statistics do not meet our criteria, and are not invariant under a change of covariate distribution. Existing methods for standardization of these statistics are either not invariant to changes in the general population mortality or are not robust. Standardized statistics and estimators are developed to address the deficiencies. They use a reference distribution for covariates such as age, and a reference population mortality survival distribution that is recommended to approach zero with increasing age as fast as the cohort with the worst life expectancy. Estimators are compared using a breast‐cancer survival example and computer simulation. The proposals are invariant and robust, and out‐perform current methods to standardize the Ederer‐II and Pohar‐Perme estimators in simulations, particularly for extended follow‐up.

## 1 Introduction

When cause of death is unavailable or unreliable it is not possible to directly estimate disease‐specific survival. For this reason, disease‐specific survival is sometimes assessed by a measure of the relative survival between a group diagnosed with disease and the wider population. The main use of relative survival analysis is to compare survival between cohorts, such as from different countries or over periods in time. A complication is that cohort structures can differ. For example, relative survival in cancer is often lower for older patients than younger patients, and different countries may have different distributions of age‐at‐diagnosis. In this article, we compare the use of relative survival measures for making such comparisons by defining general criteria based on the following setup.

Let *S* be a survival function and Λ the corresponding cumulative hazard, with superscript *C* denoting a cohort of interest (often patients diagnosed with cancer), and *P* the general population from which the cohort was derived. We assume that survival may depend on covariates *x*, including in particular age and gender. Then

$$\Lambda^{e}(t|x)=\Lambda^{C}(t|x)-\Lambda^{P}(t|x)$$

is defined to be the conditional excess cumulative hazard at time *t*, although $\Lambda^{e}$ need not be monotone or even positive. Typically *t* is time from diagnosis and $\Lambda^{P}(t|x)=\Lambda_{b}^{P}(a+t|x)$, where $\Lambda_{b}^{P}$ is the cumulative hazard from birth and *a* is the age at diagnosis. Corresponding to $\Lambda^{e}$

$$S^{e}(t|x)=S^{C}(t|x)/S^{P}(t|x)$$

is the conditional relative survival (which may not be a survival function).

The initial estimators developed by Ederer and co‐workers focused on the relative survival $E_{H}\{S^{C}(t|X)\}/E_{H}\{S^{P}(t|X)\}$, where *H* is the marginal distribution of *X*, and $E_{H}$ denotes expectations with respect to *H* (Ederer and Heise, 1959; Ederer et al., 1961). Estève et al. (1990) suggested that when $S^{e}$ depends on *x*, the target of estimation should instead be the marginal net survival

(1) $$\begin{array}{ccc} S_{\mathrm{net}}(t) & = & E_{H}\{S^{e}(t|X)\}\\ & = & \int S^{e}(t|x)dH(x). \end{array}$$

When the relative survival is homogeneous, i.e., $S^{e}(t|x)=S^{e}(t)$, then the Ederer estimators are consistent for the marginal net survival. However, Estève et al. (1990) pointed out that when the relative survival is heterogeneous the limit of the classical estimators depends on the survival in the general population *P*, and so they are not universal. They suggested modeling the excess hazard parametrically. Sasieni (1996) showed how it could be modeled semi‐parametrically, but it was not until Perme et al. (2012) that a non‐parametric estimator of the net survival corresponding to the marginal excess hazard was developed. Unlike the classical methods, the Pohar‐Perme estimator is consistent for the net survival (1) in the heterogeneous setting and, consequently, Roche et al. (2013) suggested that all classical methods should be abandoned. Lambert et al. (2015) noted a trade‐off between consistency of the new estimator and its precision.

We are not convinced that the mean of the relative conditional survival is the only statistic of interest for the comparison of survival between countries, periods in time or types of disease. Indeed, it is clear that the net survival depends on the covariate distribution, and two populations with different such distributions may have different marginal net survivals, even when the conditional net survival functions are identical. We next take a step back from focus on the net survival, by considering what features one would like a covariate‐free descriptor of the relative survival to hold.

## 2 Criteria

Consider a functional *R* of two conditional survival functions and a covariate distribution that is a function of time *t* only (i.e., *R* is not a function of covariates *x*), which describes the ratio of survival functions. For example, *R* might be the net survival:

$$R(S^{C},S^{P},H)(t)=E_{H}\{S^{C}(t|X)/S^{P}(t|X)\},$$

or it could be the relative survival:

$$R(S^{C},S^{P},H)(t)=E_{H}\{S^{C}(t|X)\}/E_{H}\{S^{P}(t|X)\}.$$

If the purpose is to recreate the ratio of survival functions when they are independent of covariates then this should be a requirement: $R(S^{C},S^{P},H)(t)=S^{C}(t)/S^{P}(t)$ whenever $S^{C}(t|x)=S^{C}(t)$ and $S^{P}(t|x)=S^{P}(t)$ for all *x*. More generally, we might require this to hold provided only that the ratio of survival functions $S^{e}$ (or equivalently the excess cumulative hazard $\Lambda^{e}$) is independent of covariates. This is our first requirement:

A1 $R(S^{C},S^{P},H)(t)=S^{e}(t)$ whenever $S^{C}(t|x)/S^{P}(t|x)=S^{e}(t)$.

$S^{e}$ may be independent of covariates in real data. For example, relative survival from advanced breast cancer in Section 6 appears to be approximately independent of age at diagnosis until t=10 years. When the ratio is not independent of covariates (A1 is vacuous, but) we would still like the statistic to reflect the ordering of the ratio.

A2a If for some *T*, $S^{C}(t|x)/S^{P}(t|x)\leq S^{C^{*}}(t|x)/S^{P^{*}}(t|x)$ for all *x* and t≤T, then $R(S^{C},S^{P},H)(t)\leq R(S^{C^{*}},S^{P^{*}},H)(t)$ for all t≤T.
A2b If for some *T*, $S^{C}(t|x)/S^{P}(t|x)=S^{C^{*}}(t|x)/S^{P^{*}}(t|x)$ for all *x* and t≤T, then $R(S^{C},S^{P},H)(t)=R(S^{C^{*}},S^{P^{*}},H)(t)$ for all t≤T.
A2c If for some *T*, $S^{C}(t|x)/S^{P}(t|x)<S^{C^{*}}(t|x)/S^{P^{*}}(t|x)$ for all *x* and t≤T, then $R(S^{C},S^{P},H)(t)<R(S^{C^{*}},S^{P^{*}},H)(t)$ for all t≤T.

Condition A2b is key for comparing relative survival between cohorts. It ensures that *R* does not depend on $S^{P}$ other than through $S^{e}$. Ideally, we would like *R* to depend on $S^{C}$ and $S^{P}$ only through their ratio even if the covariate distribution is different. This leads to our third requirement that the statistic is independent of the covariate distribution

A3 $R(S^{C},S^{P},H)(t)=R(S^{C},S^{P},H^{*})(t)$.

When both A3 and A2b are satisfied, if $S^{C}(t|x)/S^{P}(t|x)=S^{C^{*}}(t|x)/S^{P^{*}}(t|x)$ for all *x*, then $R(S^{C},S^{P},H)(t)=R(S^{C^{*}},S^{P^{*}},H^{*})(t)$. One reason for considering different statistics other than the net survival (1) is that the net survival does not satisfy A3.

Conditions A1 and A2 might be considered essential for a descriptive measure of relative survival, whereas A3 is necessary only for comparing relative survival between cohorts with different covariate distributions. By analogy, the crude rate is useful for describing a single cohort, but the age‐standardized rate is more useful when comparing two cohorts.

If a measure meets criteria A1–A3 then we might ask what additional properties would be desirable. We consider the following.

A4 Robustness. Small changes in $S^{C}$ for a fixed $S^{P}$ and *H* do not cause large changes in *R*.
A5 Precision. We prefer measures with smaller $\mathrm{var}(\hat{R})R^{-2}$, where $\hat{R}$ is an efficient estimator of *R*.

## 3 Some Relative Survival Families and Estimators

The observable data for individuals i=1,…,n are $\left(T_{i},X_{i}\right)$, where $T_{i}$ is the time of death and $X_{i}$ the covariate value; $P(T_{i}>t|x_{i})=S^{C}(t|x_{i})$, X∼H; $S_{i}^{P}(\cdot )$ is assumed known. If $\left({\hat{S}}^{C},\hat{H}\right)$ denote empirical versions of $\left(S^{C},H\right)$ (putting mass 1/n at each point $\left(T_{i},X_{i}\right)$), then corresponding to a measure $R(S^{C},S^{P},H)$ we may have an estimator $R({\hat{S}}^{C},S^{P},\hat{H})$. To allow for right censoring we follow Andersen et al. (1996) and use notation $Y_{i}(t)$ for the at‐risk process ($Y_{i}(t)=I(T_{i}\geq t)$ in the absence of censoring, where I(·) is the indicator function); and the counting process $N_{i}(t)=\int_{0}^{t}dN_{i}(u)$ where $dN_{i}(t)=Y_{i}(t)I(T_{i}=t)$.

Now, under independent censoring, consider a family of estimators of the cumulative excess hazard

(2) $$4{\hat{A}}_{w}(t)=\int_{0}^{t}\frac{\sum_{i=1}^{n}w_{i}(u)Y_{i}(u)\{dN_{i}(u)-d\Lambda_{i}^{P}(u)\}}{\sum_{i=1}^{n}w_{i}(u)Y_{i}(u)},$$

where $\Lambda_{i}^{P}$ is the cumulative hazard for an individual with covariate $x_{i}$ in the general population, and $w_{i}(t)$ is a chosen weight given to the *i*th individual at time *t*, that may depend on $x_{i}$. Setting $w_{i}(t)=1$ for all i=1,…,n and t>0 yields the Ederer‐II estimator of the cumulative excess hazard ($4{\hat{A}}_{1}$) (Ederer and Heise, 1959), and setting $w_{i}(t)={\left\{S_{i}^{P}(t)\right\}}^{-1}$ provides the Pohar‐Perme estimator ($4{\hat{A}}_{1/S^{P}}$) (Perme et al., 2012). They may be put onto a survival scale through the usual transformation $\exp\{-4{\hat{A}}_{w}(t)\}$. Since Y(u∣X)dN(u∣X)=dN(u∣X), $E\{N(u|X)|X\}=1-S^{C}(u|X)$ and $E\{Y(u|X)\}=S^{C}(u|X)$, we may write

$$4{\hat{A}}_{w}(t)=\int_{0}^{t}\frac{E_{\hat{H}}[w(u,X)\{d{\hat{S}}^{C}(u|X)-{\hat{S}}^{C}(u|X)d\Lambda^{P}(u|X)\}]}{E_{\hat{H}}\{w(u,X){\hat{S}}^{C}(u|X)\}}.$$

It follows that if the $X_{i}$ are independent and identically distributed then $4{\hat{A}}_{w}(t)$ converges to

$$\int_{0}^{t}\frac{E_{H}[w(u,X)\{dS^{C}(u|X)-S^{C}(u|X)d\Lambda^{P}(u|X)\}]}{E_{H}\{w(u,X)S^{C}(u|X)\}},$$

and because $-dS^{C}/S^{C}=d\Lambda^{C}$

$$A_{w}(t)=\int_{0}^{t}\frac{E_{H}[w(u,X)S^{C}(u,X)\{d\Lambda^{C}(u|X)-d\Lambda^{P}(u|X)\}]}{E_{H}\{w(u,X)S^{C}(u|X)\}}.$$

This leads to our first family of relative survival measures:

$$R_{w}^{1}(S^{C},S^{P},H)=\text{exp}\{-A_{w}(t)\}1\mathrm{em}\left(\mathrm{weighted}\;\mathrm{excess}\;\mathrm{hazards}\right).$$

A second family is defined

$$\begin{array}{ccc} R_{w}^{2}(S^{C},C^{P},H) & = & \frac{E_{H}\{w(t,X)S^{C}(t|X)\}}{E_{H}\{w(t,X)S^{P}(t|X)\}}\\ & & \left(\mathrm{relative}\;\mathrm{weighted}\;\mathrm{survival}\right). \end{array}$$

If w(t,X)=1 then we have the limit of the Ederer‐I estimator (Ederer et al., 1961). Relative weighted survival satisfies our criterion A1 because in this case

$$E_{H}\{w(t,X)S^{C}(t|X)\}=S^{e}(t)E_{H}\{w(t,X)S^{P}(t|X)\}.$$

In order for it to satisfy A2, and depend only on $S^{e}$ and not $S^{P}$, the weight $w(t,X)=v(t,X)/S^{P}(t)$ where v(t,X) is a weight function that does not depend on $S^{P}(t)$. Since $R_{1/S^{P}}^{2}$ is the net survival, we call $R_{v/S^{P}}^{2}$ weighted net survival.

There is a natural family of estimators corresponding to $R_{w}^{2}$ that, to our knowledge, has been not been used previously. When there is no censoring then $R_{w}^{2}$ may be estimated consistently by

(3) $$\frac{\sum_{i=1}^{n}w_{i}(t)Y_{i}^{+}(t)}{\sum_{i=1}^{n}w_{i}(t)S_{i}^{P}(t)},$$

where $Y_{i}^{+}(t)=I(T_{i}>t)$. For the more general case with censoring *D* that is independent of the covariate (so that $S_{i}^{D}=S^{D}$), we can define a family of estimators for $R_{w}^{2}$ as

(4) $$U_{w}(t)=\frac{\sum_{i=1}^{n}w_{i}(t)Y_{i}^{+}(t)}{{\hat{S}}^{D}(t)\sum_{i=1}^{n}w_{i}(t)S_{i}^{P}(t)},$$

where ${\hat{S}}^{D}(t)$ is a Kaplan–Meier estimate of the censoring survival distribution. When w(t,x)=1 we have Ederer‐I and when $w(t,x)=1/S^{P}(t|x)$ we have an alternative to the Pohar‐Perme estimator that is also consistent for the net survival. Further, when there is no competing mortality so that $S_{i}^{P}(t)=1$ for all *i* and *t*, then ${\hat{R}}_{1}={\hat{R}}_{1/S^{P}}$ and $U_{1}=U_{1/S^{P}}$, and it can be shown that $U_{1}(t)={\hat{R}}_{1}^{1}(t)$, with both equal to the Kaplan–Meier estimate of $S^{C}(t)$ in the cohort (which is the non‐parametric maximum likelihood estimate).

We end by imposing some restrictions on the weights w(t,x) based on the criteria. By definition the first two arguments to each *R* may be stated in terms of any two of $S^{C}$, $S^{P}$, and $S^{e}$. If we consider R as a function of $\left(S^{P},S^{e},H\right)$, then A2a and A3 imply that it depends only on $S^{e}$. Suppose that $R_{w^{\prime }}$ satisfied A2a and A3 and that $w=w^{\prime }v$, then for $R_{w}$ to also satisfy A2a and A3 *v* should depend on $\left(S^{P},S^{e},H\right)$ only via $S^{e}$.

## 4 Assessment of Criteria

We next consider whether the families $R_{w}^{1}$ and $R_{w}^{2}$ satisfy our fundamental requirements A1–A3.

A1 Both $R_{w}^{1}$ and $R_{w}^{2}$ satisfy A1. This is seen by taking the excess terms such as $S^{e}$ outside of the expectation.
A2 When $w(t,x)=v(t,x)/S^{P}(t|x)$ and v(t,x) depends on $\left(S^{C},S^{P},H\right)$ only through $S^{e}$ (or not at all) then both $R_{w}^{1}$ and $R_{w}^{2}$ satisfy A2. It is for this reason that the limit of the Ederer‐I and II estimators do not satisfy A2: they depend on $S^{P}$ even when $S^{e}$ is fixed.
A3 Neither $R_{w}^{1}$ nor $R_{w}^{2}$ are guaranteed to satisfy A3. In order for them to do so one needs to standardize so that the weights are proportional to the ratio of the standardized to the observed covariate density, i.e., $h^{0}(x)/h(x)$, using the superscript 0 to denote a standard population. This is the approach to age adjustment that was proposed by Brenner et al. (2004); see Section 5.2 for further discussion. If we wish to standardize two cohorts with covariate distributions *H* and $H^{*}$ that do not have the same support, then to meet A3 the support of the standard distribution $H^{0}$ should be their intersection only (i.e., $h^{0}(X)=0$ if either h(X)=0 or $h^{*}(X)=0$).

We have, thus, established that $R_{w}^{1}$ and $R_{w}^{2}$ satisfy our main requirements A1–A3 provided $w(t,x)=h^{0}(x)v(t,x)/\{S^{P}(t|x)h(x)\}$ and v(t,x) depends on $\left(S^{C},S^{P},H\right)$ only via $S^{e}$.

A4 Assuming $w=h^{0}v/({\mathrm{hS}}^{P})$ then $R_{w}^{1}(t)$ becomes
  $$\text{exp}\left[-\int_{0}^{t}\frac{E_{H^{0}}\{v(u,X)S^{e}(u|X)d\Lambda^{e}(u|X)\}}{E_{H^{0}}\{v(u,X)S^{e}(u|X)\}}\right],$$
  and $R_{w}^{2}(t)$ becomes
  $$\frac{E_{H^{0}}\{v(t,X)S^{e}(t|X)\}}{E_{H^{0}}\{v(t,X)\}}.$$
  It is then clear that in order for $R_{w}^{1}$ and $R_{w}^{2}$ to be robust (against for instance a very large |d$\Lambda^{e}(u|X_{i})|$ which might happen in a sample when $S^{C}$ is very small), one should require that w(u,x) is bounded for all *u* and *x*. When $w=h^{0}v/({\mathrm{hS}}^{P})$ then this can either be achieved by setting $h^{0}(x)=0$ when $S^{P}$ is very small (compared with other *x* at the same *t*) or ensuring that $v/S^{P}$ is bounded. Further, consider $S^{C\Delta }$ such that $|S^{C\Delta }(t|x)-S^{C}(t|x)|\leq \Delta$ for all *t* and *x* and $S^{C\Delta }(t|x)=S^{C}(t|x)-\Delta$ for $t_{l}<t<t_{u}$, some *x* and small constant Δ>0 where $S^{C}(t_{*}|x)=\varepsilon >\Delta$ for some $t_{*}\in (t_{l},t_{u})$. Then assuming the hazards exist
  $$\begin{array}{ccc} \lambda^{C}(t_{*}|x) & = & -\frac{dS^{C}(t_{*}|x)/dt}{\varepsilon }\\ \lambda^{C\Delta }(t_{*}|x) & = & -\frac{dS^{C}(t_{*}|x)/dt}{\varepsilon -\Delta } \end{array}$$
  and
  $$\frac{\lambda^{C\Delta }(t_{*}|x)}{\lambda^{C}(t_{*}|x)}=\frac{\varepsilon -\Delta }{\varepsilon }.$$
  Thus, for fixed Δ, as *t* gets large so that $S^{C}(t|x)=\varepsilon$ gets small, $\lambda^{C\Delta }(t|x)$ may be substantially different from $\lambda^{C}(t|x)$, which affects the excess hazard (for fixed $S^{P}$). In other words, $R_{w}^{1}$ and $R_{w}^{2}$ are not robust unless *w* is carefully chosen: for each *x*, the weights $w(t,x)S^{C}(t,x)/E_{H}\{w(t,X)S^{C}(t|X)\}$ need to approach zero with *t* as fast or faster than $S^{P}(t|x)$. This argument is also relevant for comparisons between populations: to be robust $w(t,x)/E_{H}\{w(t,x)\}$ should approach zero as fast or faster than $S^{P}(t|x)$ in all populations compared. Recall that if *a* is age at diagnosis, then $S^{P}(t|a)=S_{b}^{P}(t+a)$ where $S_{b}^{P}(t)$ is the probability of living until age *t* in the general population.
A5 The asymptotic variance of ${\hat{R}}_{w}^{1}$ may be estimated by ${\hat{R}}_{w}^{1}(t){\hat{\sigma }}^{2}(t)$ using the same arguments as Perme et al. (2012) where
  (5) $$\begin{array}{ccc} {\hat{\sigma }}^{2}(t) & = & \int_{0}^{t}\frac{J(u)\sum_{i=1}^{n}dN_{i}(u)w_{i}^{2}(u)}{{\left\{\sum_{i=1}^{n}Y_{i}(u)w_{i}(u)\right\}}^{2}}, \end{array}$$
  with $J(t)=I\{\sum_{i=1}^{n}Y_{i}(t)>0\}$.
  The variance of the estimator of $R_{w}^{2}(t)$ in (3) is
  (6) $$\frac{\sum_{i=1}^{n}w_{i}^{2}S_{i}^{C}(1-S_{i}^{C})}{{\left(\sum_{i=1}^{n}w_{i}S_{i}^{P}\right)}^{2}}.$$
  It is not straightforward to use this formula for estimation because of the difficulty in estimating $S_{i}^{C}$ without modeling its dependence on *X*. Although E$\left\{Y_{i}^{+}(t)\}=S_{i}^{C}(t\right)$, $Y_{i}^{+}(t)\in \{0,1\}$ so we cannot in general simply replace $S_{i}^{C}$ in (6) by $Y_{i}^{+}$. One exception is when there are assumed to be j=1,…,k homogeneous groups of size $n_{j}$. Then, with independent censoring within each strata one may estimate $R_{w}^{2}$ as
  $$\frac{\sum_{j=1}^{k}n_{j}w_{j}(t){\hat{S}}_{j}^{C}(t)}{\sum_{j=1}^{k}n_{j}w_{j}(t)S_{j}^{P}(t)},$$
  based on a stratum‐specific Kaplan–Meier estimate ${\hat{S}}_{j}^{C}(t)$, and the variance may be estimated via
  $$\frac{\sum_{j=1}^{k}n_{j}w_{j}^{2}\mathrm{var}({\hat{S}}_{j}^{C})}{{\left(\sum_{j=1}^{k}n_{j}w_{j}S_{j}^{P}\right)}^{2}},$$
  where Greenwood's formula might be used for $\mathrm{var}({\hat{S}}_{j}^{C})$. However, in practice a bootstrap estimate of the variance of $U_{w}$ is recommended because one may avoid the assumption of homogeneous groups.
  Precision of the estimators of $R_{w}^{1}$ and $R_{w}^{2}$ is clearly affected by the choice of weight function due to the $w_{i}^{2}$ term in the numerator of the variance. In both, functions that place more weight on the oldest patients, such as $w_{i}(t)=1/S_{i}^{P}(t)$ (Pohar‐Perme with $R_{w}^{1}$) are less precise than others with weights such as $w_{i}(t)=1$ (Ederer‐II with $R_{w}^{1}$), or $w_{i}(t)=v_{i}(t)/S_{i}^{P}(t)$ where $v_{i}(t)$ down‐weights small $S_{i}^{P}(t)$.

## 5 Standardization

Methods of standardization that are used in the numerical sections of this article are next introduced, and discussed in relation to the criteria A1–5.

### 5.1 Stratification

The Ederer‐II and Pohar‐Perme estimators are often standardized by stratification, particularly by age group (Pokhrel and Hakulinen, 2008). The most common method is a weighted arithmetic mean of stratum‐specific estimates of the relative survival ${\hat{R}}_{j}$ in stratum j=1,…,k. Let $g_{j}=P_{H^{0}}(x_{i}\in G_{j})$ for groups $G_{j}$. Then denote the traditional standardized statistic by

(7) $$\begin{array}{ccc} D_{g}(R) & = & \sum_{j=1}^{k}g_{j}R_{j}. \end{array}$$

$D_{g}$ satisfies A1–A3 provided the statistic $R_{j}$ satisfies A1–A3 in each stratum. Note also that when the same level of stratification is used for the weights in ${\hat{R}}_{w}^{1}$ and for weights in the standardization, (i.e., if $w_{i}(t)=w_{i^{\prime }}(t)$ whenever the observations *i* and $i^{\prime }$ come from the same stratum $G_{j}$), then $D_{g}({\hat{R}}_{w}^{1})$ does not depend on the particular weights since the $w_{i}(u)$ terms in (2) cancel out. Thus, when the same factors are used to stratify the population mortality $S^{P}$ and for standardization by stratification, the standardized Ederer‐II (corresponding to $w_{i}=1$) and the standardized Pohar‐Perme (corresponding to $w_{i}=1/S_{i}^{P}$) estimators will be identical.

### 5.2 Baseline Weighting

A problem with stratification is that the number in each stratum needs to be sufficient to obtain an estimate of $S^{e}$ over the follow‐up period of interest: with censored data it is not possible to estimate the excess survival beyond the smallest of the stratum‐specific last follow‐up times. A second approach to standardization is to use a weighted estimator. Each individual is weighted so that the weighted sample at baseline represents the standard population (Brenner et al., 2004). This approach corresponds to using time‐constant weights within the estimator, rather than taking a weighted average of stratum‐specific estimates. It is exactly what needs to be done to ensure that our condition A3 is satisfied. When used with Ederer‐II one has weights $(nz_{i})/n_{i}$, where $n_{i}=\sum_{j=1}^{n}I(x_{j}=x_{i})$ is the number of individuals in the sample at baseline with the same covariate values, and $z_{i}$ is a standard probability mass function for the covariates ($\sum_{i=1}^{n}z_{i}=1$). When this approach is applied to the Pohar‐Perme estimator one has

(8) $$\begin{array}{ccc} w_{i}^{B}(t) & = & \frac{nz_{i}}{S_{i}^{P}(t)n_{i}}. \end{array}$$

Unlike the usual Pohar‐Perme estimator these weights satisfy A3 for both ${\hat{R}}_{w}^{1}$ and ${\hat{R}}_{w}^{2}$, but they are similarly not robust.

### 5.3 Standardized Relative Survival

Our proposal is to standardize the estimators of $R_{w}^{1}$ and $R_{w}^{2}$ by using weights

(9) $$\begin{array}{ccc} w_{i}(t) & = & \frac{nz_{i}S_{i}^{0}(t)}{S_{i}^{P}(t)n_{i}}. \end{array}$$

If the covariate distribution at time 0 is $z_{i}$ and individuals are subject to survival $S_{i}^{0}(t)$, then $z_{i}S_{i}^{0}(t)$ will be the covariate distribution at time *t* in the standard population. Thus, $z_{i}S_{i}^{0}(t)$ can be thought of as a standard prevalence of patients with the disease and covariates $x_{i}$ at time *t* post diagnosis. The limit of the estimators with these weights corresponds to $R_{w}^{1}$ and $R_{w}^{2}$ with $w=h^{0}S^{0}/(S^{P}h)$. With these weights $R_{w}^{1}$ and $R_{w}^{2}$ meet A3 as described above. The parameterization is still arbitrary, in that $S^{0}$ and $H^{0}$ (or $z_{i}$) may be chosen, but A4 helps to rule out certain choices of $S^{0}$. For example, if $H^{0}=H$ and $S^{0}(t)=1$ for all *t* then $R_{w}^{1}$ is the Pohar‐Perme estimator which does not meet A4. Suppose that X=(a,l) where *a* is age at diagnosis and *l* is a categorical variable with l=1,2,…,L levels, and $S^{P}(t|a,l)=S_{\mathrm{bl}}^{P}(a+t)$, where $S_{\mathrm{bl}}^{P}$ is the survival from birth in group *l*. Then to meet A4 we showed that the standard reference weights should be chosen so that $S_{\mathrm{bl}}^{0}(t)\leq S_{\mathrm{bl}}^{P}(t)$ for all *l* and *t*. For instance, a country with the poorest population survival could provide $S^{0}$.

Equations (5) and (6) show how the choice of $S^{0}$ in (9) relates to A5. The proposed weight $w_{i}(t)=h^{0}(x_{i})S_{i}^{0}(t)/\{h(x_{i})S_{i}^{P}(t)\}$ enables us to ensure $S_{i}^{0}(t)/S_{i}^{P}(t)$ is stable through the choice of $S^{0}$. Equations (5) and (6) also show that there may be a trade‐off between robustness and precision. If $w_{i}(t)$ is zero then the data from individual *i* will not be used for estimation at time *t*; this will give the estimator robustness against outlying events at time *t*, but (5) will be larger and precision worse. Thus, one would not wish to set $S^{0}(t|x)$ to zero for t≥T unless there is no interest in estimating *R* at or beyond time *T*.

In summary, we have two measures and corresponding estimators that satisfy criteria A1–A4, under an assumption of independent censoring. It is not clear whether there are circumstances when one might dominate the other in terms of estimation precision (A5). This will be explored later using a computer simulation.

To help interpretation note that when $S^{0}=S^{P}$ and $h^{0}=h$, the weights in (9) equal one. Thus, $R_{w}^{2}$ and $R_{w}^{1}$ are, respectively, the Ederer‐I and II estimation targets when the standard survival is taken to be the same as that in the reference population, and the standard covariate distribution is the same as in the observed cohort. Suppose instead that $S^{P}=1$, i.e., there is no competing hazard) then both Ederer‐II and Pohar‐Perme weights in $R_{w}^{1}$ are one, and ${\hat{R}}_{w}^{1}$ gives the Kaplan–Meier estimator (more precisely $4{\hat{A}}_{w}$ gives the Nelson–Aalen estimator). The use of $S^{0}$ in our weights (9) provides a stratum‐weighted Kaplan–Meier estimator (Xie and Liu, 2005). Thus, $R_{w}^{1}$ with weights given by (9) can be interpreted as the marginal net survival that would be observed in population $H^{0}$ subject to censoring $S^{0}(t|x)$. It might be called the $S^{0}$‐filtered net survival. At each time *t*, $R_{w}^{1}$ corresponds to a weighted average of the conditional excess hazard functions: E${}_{H^{0}}\{w(t,X)\lambda^{e}(t|X)\}$, where $E_{H^{0}}\{w(t,X)\}=1$. If the excess hazard is independent of *X* then the weights do not matter. More generally we want the weights to be reasonably homogeneous. In particular, we would like to give (approximately) equal weight to subsets of *X* that have equal probability of being at risk at time *t*. Ederer‐II does this exactly but at a price—it does not satisfy A2. Our weights (9) provide a good approximation to homogeneous weighting while ensuring A1–A3 hold.

## 6 Example

The R package relsurv (Perme, 2013), which implements the Pohar‐Perme, Ederer‐II, and some other relative survival estimators was extended to fit the standardized methods in this report (supplementary material). To demonstrate the methods, we obtained data on breast cancers diagnosed between 1973 and 2010 in the United States from SEER (2014). Death rates for the same period were obtained from National Center for Health Statistics (2015) by age and gender. The following reference data were used for standardization.

1. The reference age distribution of cases was a standard taken from Corazziari et al. (2004). This weights age groups (15–44, 45–54, 55–64, 65–74, 74+) as (7, 12, 23, 29, 29)%.
2. For exposition the standard reference mortality rate was taken to be that estimated for the Russian Federation (Human Mortality Database, 2016), where mortality rates were approximately 70% higher than in the United States for women aged 60 between 1980 and 1989, rising to 300% by 2000–2010. The effect of a lower reference rate (not recommended) was considered by dividing the U.S. mortality rates by three.

We focus on the survival of 16,597 women younger than 85 who were diagnosed with invasive breast cancer with distant spread (stage 4 based on SEER historic stage A), between 1980 and 2010, of whom 15,572 died after a median follow‐up of 7.9 years.

**Figure 1:**
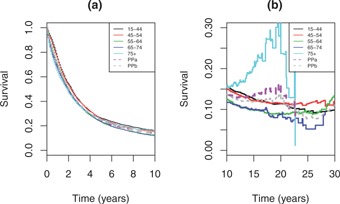
Pohar‐Perme net survival estimates by age band and age‐standardized: (a) to 10 years; (b) beyond 10 years. PPa, traditional age‐standardization; PPb, age standardization based on (8).

Figure 1 shows the Pohar‐Perme relative survival estimates by age band, where there was little difference to 10 years between the younger (<55) and older groups. Thus, to 10 years, net survival appeared to be almost independent of age at diagnosis (*c.f*. criteria A1). However, beyond 10 years the differences become more pronounced for the 75+ group, as competing mortality rates increased and precision decreased. This had an impact on the traditional age‐standardization estimate, as this age group is weighted most heavily.

**Figure 2:**
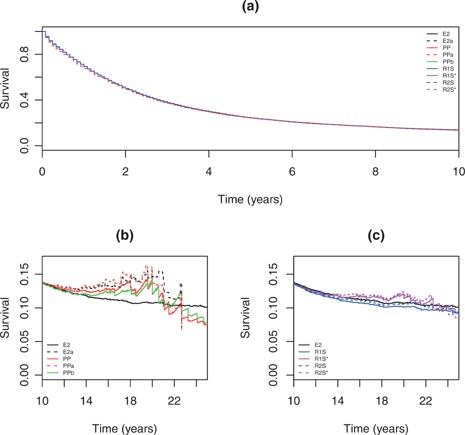
Estimated relative survival curves from example: (a) to 10 years; and beyond 10 years for (b) some existing methods and (c) proposed estimators with reference to Ederer‐II. E2, Ederer‐II estimate; PP, Pohar‐Perme estimate; PPa, E2a, traditional age‐standardization from (7); PPb, Brenner age standardization from (8); R1S, proposal ${\hat{R}}_{w}^{1}$ with (9) and standard reference mortality from the Russian Federation; R1S*, as R1S but with standard mortality rates three times lower than the United States; R2S, proposal estimated by ${\hat{U}}_{w}$ from (4) with standard rates from the Russian Federation; R2S*, similarly but with standard rates three times lower than the United States.

Figure 2 compares un‐standardized and standardized estimates. To 10 years where there was very little difference in net survival by age, there was very little practical difference between the estimators; only a very small difference is visible between the stratified estimators and the others. Larger differences were seen after 10 years. Traditional age‐standardization of Pohar‐Perme or Ederer‐II yielded very similar estimates, with substantial variability. The Brenner age‐adjustment of Pohar‐Perme was close to un‐standardized Pohar‐Perme. Our proposals (with a reference mortality that is higher than in the United States) were less variable and closer to un‐standardized Ederer‐II than the others. Reference rates lower than the United States are only shown for insight and are not recommended; as expected these weights yield an estimate with properties somewhere between those of the Pohar‐Perme and Ederer‐II estimates.

## 7 Two‐Group Simulation

A computer simulation with the following characteristics was used to further compare the estimators. Mortality rates in cohort 1 were based on women in the United States in 1980, in cohort 2 they were (i) 1.2 times higher for those younger than 70, (ii) two times higher for those aged 70–85, and (iii) four times higher when aged 86 or older. The standard reference population rates were (i) two times higher for those younger than 70, (ii) four times higher for those aged 70–85, and (iii) 100 times higher when aged 86 or older, to reflect a standard population where very few people lived into their 90s. The excess hazard was the same in both cohorts, being 3% greater per year from age 65. There were two groups in each population aged 65 or 75 at diagnosis. The percentage aged 65 at diagnosis was 60% for cohort 1, 70% for cohort 2, and 50% in the standard reference population. There were two censoring scenarios: (i) no censoring, and (ii) uniform censoring between 1 and 25 years. In the first cohort, approximately 41% were censored before their event time, and 35% in the second cohort. The outcomes of interest were estimates of relative survival at 5, 10, 15, and 20 years. A group of 2000 individuals was simulated 5000 times from both cohort populations.

Standardization is needed or methods will show a difference between the cohorts that only reflects their age distribution at baseline; we used the methods from Section 5.

**Figure 3:**
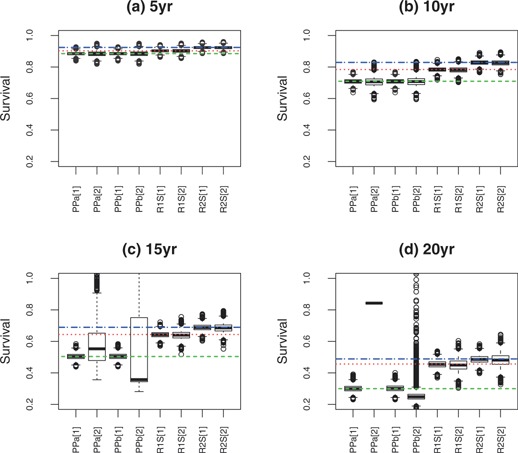
Estimated standardized relative survival from simulation example at: (a) 5, (b) 10, (c) 15, and (d) 20 years. The true net (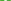) and standard survival statistics (··· $R_{w}^{1}$, 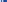 $R_{w}^{2}$; both with weights (9)) in the reference population are given; samples are from two cohort populations [1] and [2]. Net survival estimates are from PPa, traditional standardization applied to Pohar‐Perme estimation, and PPb which is Brenner standardization from (8). The standardized survival estimates $R_{w}^{1}$ and $R_{w}^{2}$ with weights (9) are labeled, respectively, R1S and R2S.

We focus first on the simulations without censoring. Figure 3 shows boxplots of the simulation survival estimates, and summary statistics are given in Table 1. The plots highlight that the net and standardized survival in the reference population are different quantities. Our interest is not in a comparison of how well the estimator for standardized survival recovers net survival etc, but whether one would draw an appropriate conclusion when comparing the two cohorts. For this the plots show little difference to 10 years. All the estimators had only small bias, and the right conclusion would be drawn for all the estimators. However, at 5 and 10 years, standardized relative survival $R^{1}$ and $R^{2}$ were more precise than net survival in terms of Var($\hat{R}$)$R^{-2}$ (Table 1), so they would rule out larger differences because they are more precise.

**Table 1:** Summary results from simulation. Net survival estimates are from PPa, traditional standardization (7) applied to Pohar‐Perme estimation; PPb, Brenner standardization of Pohar‐Perme estimation from (8); R1S, standardized survival based on (9); R2S, standardized survival based on (9); [1], first cohort; [2], second cohort; SD, standard deviation of estimates $\hat{R}$, i.e., $\sqrt{\mathrm{var}(\hat{R})}$; ****results could not be estimated in some simulations, so summary statistics excluded

	Without censoring			With independent censoring		
	Mean	SD	Var($\hat{R}$)$R^{-2}$	Mean	SD	Var($\hat{R}$)$R^{-2}$
	bias (%)	(×100)	(×10000)	bias (%)	(×100)	(×10000)
(a) 5‐yr						
PPa[1]	−0.1	1.1	1.7	0.0	1.2	1.7
PPb[1]	0.0	1.1	1.7	0.0	1.2	1.7
R1S[1]	0.0	1.0	1.2	0.0	1.0	1.3
R2S[1]	−0.1	0.9	1.0	−0.1	1.0	1.2
PPa[2]	−0.1	1.7	3.7	−0.2	1.8	4.0
PPb[2]	−0.1	1.7	3.7	−0.1	1.8	4.0
R1S[2]	0.0	1.3	2.1	−0.1	1.3	2.2
R2S[2]	−0.1	1.0	1.3	−0.1	1.1	1.4
(b) 10‐yr						
PPa[1]	−0.1	1.7	5.5	−0.1	1.9	7.4
PPb[1]	−0.1	1.7	5.5	0.0	1.9	7.5
R1S[1]	−0.1	1.5	3.7	0.0	1.7	4.7
R2S[1]	−0.2	1.6	3.7	−0.1	2.1	6.3
PPa[2]	−0.5	2.9	16.4	−0.5	3.3	21.2
PPb[2]	−0.3	2.9	16.5	−0.3	3.3	21.5
R1S[2]	−0.3	1.9	6.0	−0.3	2.1	7.4
R2S[2]	−0.4	1.9	5.2	−0.3	2.2	7.2
(c) 15‐yr						
PPa[1]	0.0	2.0	15.8	0.0	2.6	26.6
PPb[1]	0.1	2.0	15.9	0.2	2.6	27.4
R1S[1]	−0.2	2.0	9.4	−0.1	2.5	15.5
R2S[1]	−0.3	2.1	9.6	−0.1	3.1	19.7
PPa[2]	****	****	****	****	****	****
PPb[2]	0.5	25.6	2586.3	−0.6	35.5	4955.4
R1S[2]	−0.6	2.7	17.9	−0.6	3.5	30.0
R2S[2]	−0.5	2.9	17.5	−0.6	3.8	30.7
(d) 20‐yr						
PPa[1]	0.3	2.1	47.8	****	****	****
PPb[1]	0.7	2.1	49.6	0.7	3.8	161.2
R1S[1]	−0.5	2.3	25.9	−0.5	4.0	76.0
R2S[1]	−0.6	2.5	26.1	−0.7	4.5	83.6
PPa[2]	****	****	****	****	****	****
PPb[2]	−11.9	23.1	5948.3	0.4	76.5	65030.4
R1S[2]	−1.0	3.8	69.9	−1.3	6.4	198.1
R2S[2]	−1.1	4.1	69.8	−1.6	6.9	201.5

Beyond 10 years the net survival estimators started to break down, showing differences between the cohorts even though the age‐specific excess hazards are identical. This is reflected by substantial differences between estimates of net survival in the second cohort compared with the first. The reason accounts for the lack of results for traditional standardization at 20‐years survival, where it was not possible to estimate relative survival in the second cohort because everyone in the older group was dead. This robustness issue likewise affected the Brenner baseline standardization method. Our standardization methods performed robustly even at 20 years, as the standard reference population effectively excluded everyone once they were older than 85.

Censoring decreased the precision of all estimators, but did not appear to affect ${\hat{R}}_{w}^{1}$ very much more than ${\hat{R}}_{w}^{2}$. To 5 years ${\hat{R}}_{w}^{1}$ had slightly better precision than ${\hat{R}}_{w}^{2}$. Beyond that it was very similar to ${\hat{R}}_{w}^{1}$ for no censoring, and slightly worse with censoring in cohort 2: there was not a clear winner between ${\hat{R}}_{w}^{1}$ and ${\hat{R}}_{w}^{2}$ in these simulations.

## 8 Discussion

In this article, we outlined some criteria for relative survival, and then assessed different families of measures. We developed two new measures and estimators that met our criteria. Standardized $R^{1}$ may be interpreted as the marginal net survival that would be “observed” in a standard population subjected to standard censoring. This is because it provides the survival transform of a weighted excess hazard: viewing the weights as the probability of being at risk in the standard population (at time *t* given covariate *x*) gives the interpretation (provided that $S^{C}(t)\leq S^{P}(t)$). Standardized $R^{2}$ targets a marginal probability of surviving the excess hazard from the disease if the person would survive as long with respect to the standard population. It has a similar interpretation to the stratified standardization approach of Brenner and Hakulinen (2003), who proposed time‐dependent weights of the form $S_{j}^{0}(t)$ in the context of stratified estimation (Pokhrel and Hakulinen, 2008). Here, we applied similar weights but to the individual subject, which follows the ideas in Brenner et al. (2004), also incorporating the inverse probability of sampling weights introduced in this context by Perme et al. (2012). Interpretation of $R_{w}^{1}$ is arguably easier than $R_{w}^{2}$, because the excess (non‐cumulative) hazard and relative density functions corresponding to $R_{w}^{1}$ do not depend on the derivative of the weights with respect to time dw/dt, whereas for $R_{w}^{2}$ they do depend on the derivative dw/dt. However, both are statistical constructs. For a non‐specialist audience we suggest to describe both proposals as standardized relative survival indicies designed to accurately and precisely determine the direction and ordering of survival differences between cohorts.

Standardized $R^{1}$ and $R^{2}$ may be applied for longer follow‐up than traditional standardization, by placing more weight on those young enough to be expected to survive that long after diagnosis. But, they are not consistent estimators of the marginal net survival. In our view, this is much less important than our other criteria. Indeed, whenever one uses a Pohar‐Perme estimator that is standardized by stratification, one is already foregoing having an estimator of the (unstandardized) net survival. More importantly, any measure of relative survival that is not the same when the excess hazard given covariates is the same in two populations, seems more deficient than one which is inconsistent for estimation of the marginal net survival. We do not accept the need to only estimate the marginal net survival, and would prefer to precisely estimate the mean net survival with respect to a standard covariate distribution. Our argument mirrors Bickel and Lehmann (1975), who showed that although a trimmed mean is not an unbiased estimate of the mean of an asymmetric distribution, it has a place as a measure of central location of a distribution, and may be better for this than the mean in many situations.

This article has considered properties of relative‐survival measures and estimators, and from this some general guidance was provided on how to choose the standardization weights. More practically, it would be useful to provide investigators recommended tables of standard weights. We will develop elsewhere recommended cancer‐site specific standardization tables for our methods. Another limitation is that we have not considered dependent censoring patterns, such as those described by, Hakulinen (1982); Kodre and Perme (2013). Future work will address this and testing differences between standardized relative survival estimates.

In conclusion, we hope that the criteria developed to assess relative survival measures and estimators are useful for a theoretical understanding of their properties. We recommend that our proposed standardization methods be considered for non‐parametric relative survival estimation, when the aim is to make comparisons between cohorts, such as from different countries or periods in time, or even between disease types.

## 9 Supplementary Materials

An R package implementing the new methods is available with this article at the *Biometrics* website on Wiley Online Library.

## Supporting information

Supplementary Materials Code.
