# Supplementary material for: On standardized relative survival
Source: Biometrics. 2016 Aug 23;73(2):473–82. doi: 10.1111/biom.12578 (PMC5507182; doi:10.1111/biom.12578)
Supplement: Supplementary file 1 — Supplementary Materials Code. [file BIOM-73-473-s001.gz › suppcode/package/relsurvs/html/00Index.html]

R: Standardized relative survival

# Standardized relative survival

---

## Documentation for package ‘relsurvs’ version 0.0-1

- DESCRIPTION file.

## Help Pages

|  |  |
| --- | --- |
| cmp.rel | Compute crude mortality curves |
| epa | Excess hazard function smoothing |
| invtime | Inverse transforming of time in Relative Survival |
| joinrate | Join ratetables |
| plot.cmp.rel | Compute crude mortality curves |
| plot.rs.br | Test the Proportional Hazards Assumption for Relative Survival Regression Models |
| plot.rs.surv | Compute a Relative Survival Curve |
| plot.rs.surv | Compute a Relative Survival Curve |
| plot.rs.zph | Graphical Inspection of Proportional Hazards Assumption in Relative Survival Models |
| print.cmp.rel | Compute crude mortality curves |
| print.rs.br | Test the Proportional Hazards Assumption for Relative Survival Regression Models |
| rdata | Survival Data |
| residuals.rsadd | Calculate Residuals for a "rsadd" Fit |
| rs.br | Test the Proportional Hazards Assumption for Relative Survival Regression Models |
| rs.surv | Compute a Relative Survival Curve |
| rs.surv.rsadd | Compute a Relative Survival Curve from an additive relative survival model |
| rs.survw | Compute a Relative Survival Curve |
| rs.zph | Behaviour of Covariates in Time for Relative Survival Regression Models |
| rsadd | Fit an Additive model for Relative Survival |
| rsmul | Fit Andersen et al Multiplicative Regression Model for Relative Survival |
| rstrans | Fit Cox Proportional Hazards Model in Transformed Time |
| slopop | Census Data Set for the Slovene Population |
| survfit.rsadd | Compute a Predicited Survival Curve |
| survsplit | Split a Survival Data Set at Specified Times |
| transrate | Reorganize Data into a Ratetable Object |
| transrate.hld | Reorganize Data obtained from Human Life-Table Database into a Ratetable Object |
| transrate.hmd | Reorganize Data obtained from Human Mortality Database into a Ratetable Object |
